# Microstructure and Mechanical Behavior Comparison between Cast and Additive Friction Stir-Deposited High-Entropy Alloy Al_0.35_CoCrFeNi

**DOI:** 10.3390/ma17040910

**Published:** 2024-02-16

**Authors:** Zackery McClelland, Kyle Dunsford, Brady Williams, Haley Petersen, Keivan Devami, Mark Weaver, J. Brian Jordan, Paul G. Allison

**Affiliations:** 1 US Army Engineer Research and Development Center, Vicksburg, MS 39180, USA; 2 General Electric Aerospace, Cincinnati, OH 45215, USA; 3 Department of Mechanical Engineering, Baylor University, Waco, TX 76706, USA; 4 Department of Mechanical Engineering, Mississippi State University, Starkville, MS 39762, USA; 5 Department of Mechanical Engineering, The University of Alabama, Tuscaloosa, AL 35487, USA; 6 Department of Metallurgical Engineering, The University of Alabama, Tuscaloosa, AL 35487, USA

**Keywords:** AlCoCrFeNi HEA, additive friction stir deposition, microstructure, mechanical property, strength

## Abstract

High-entropy alloys (HEAs) are new alloy systems that leverage solid solution strengthening to develop high-strength structural materials. However, HEAs are typically cast alloys, which may suffer from large as-cast grains and entrapped porosity, allowing for opportunities to further refine the microstructure in a non-melting near-net shape solid-state additive manufacturing process, additive friction stir deposition (AFSD). The present research compares the microstructure and mechanical behavior of the as-deposited AFSD Al_0.35_CoCrFeNi to the cast heat-treated properties to assess its viability for structural applications for the first time. Scanning electron microscopy (SEM) revealed the development of fine particles along the layer interfaces of the deposit. Quasi-static and intermediate-rate compression testing of the deposited material revealed a significant strain-rate sensitivity with a difference in yield strength of ~400 MPa. Overall, the AFSD process greatly reduced the grain size for the Al_0.35_CoCrFeNi alloy and approximately doubled the strength at both quasi-static and intermediate strain rates.

## 1. Introduction

Solid solution high-entropy alloys (HEAs) and related multiphase complex concentrated alloys (CCAs) have been receiving considerable attention in the scientific community due to their often-unique microstructures and properties. Thus, HEAs are being considered for several applications, including as high-temperature dies and molds, turbine blades, thermal spray bond coatings, and in renewable energy due to their mechanical properties, such as hardness, strength, ductility, fatigue, and fracture toughness, far exceeding those of traditional alloys [1,2,3,4,5,6]. These alloys generally contain three to five principal elements in equal or near-equal molar quantities, which imparts them with high mixing configurational entropies which are thought to promote the formation of high-symmetry solid solution phases [7,8,9,10].

Presently, mechanical testing on HEAs is still in the early stages and has been mainly focused on the deformation behavior of HEAs at quasi-static strain rates and high temperatures [8,11,12,13]. A significant amount of work has also been invested in understanding the benefits of FCC and BCC phases to strength and ductility in various HEA materials, but this research was limited to only quasi-static strain rates [14,15,16,17,18]. The literature shows that single-phase FCC materials are typically more ductile, while single-phase BCC materials display higher strengths; however, dual-phase FCC + BCC microstructures display a mix of both properties [19,20]. However, many of the investigated alloys contained more than just FCC and BCC phases. As-cast AlCoCrFeNiTi, for example, has been shown to consist of BCC and B2 phases with a yield strength, fracture strength, and plastic strain limit of 2.26 GPa, 3.14 GPa, and 23.3%, respectively [8].

Currently, there is limited work on how these solid solution materials behave under various processes that change the established cast microstructure, with most of the work on the additive space, focusing on laser powder bed fusion (LPBF) or directed energy deposition (DED) [21,22,23,24,25]. Severe plasticity, through thermomechanical processes such as additive friction stir deposition (AFSD) and friction stir welding (FSW), has been shown to develop fine nanocrystalline or near-nanocrystalline grain structures with increased performance in a number of materials, including pure Cu and IN625 [26,27]. Similarly, Avery et al. observed a 97% reduction in grain size in AA7075 subjected to AFSD [28]. Process–structure–property studies by Rivera et al. revealed similar reductions in grain size in AA2219 and Phillips et al. observed a 93% reduction in grain size in AA6061 subjected to AFSD [26,29]. Rivera et al. also observed a reduction in grain size from 30 μm to 0.5 μm in an IN625 alloy [26]. Dynamic recrystallization (DRX) has been attributed as the main cause for grain refinement in AFSD and could potentially lead to significant strength gains through Hall–Petch strengthening of the deposited materials [30]. However, little work has been performed on the AFSD of HEA materials and if DRX would provide similar improvements. Agrawal et al. observed a significant reduction in the grain size of an Fe_40_Mn_20_Co_20_Cr_15_Si_5_ (at%) HEA subjected to AFSD due to discontinuous dynamic recrystallization. Agrawal et al. also observed an increase in the grain size from alterations in the processing parameter increasing the heat generated and subsequently affecting the mechanical properties [31]. However, all mechanical characterization work was performed at quasi-static rates and there is still limited understanding of strain rate sensitivity.

A side effect of aluminum alloys subjected to AFSD, however, is the refinement of intermetallics [29,32,33]. How these intermetallics could affect the overall deformation behavior of an HEA is of great interest as a potential extra strengthening mechanism. Currently, solid solution strengthening is the main mechanism for increased strength in HEAs and adding other strengthening mechanisms or creating multi-phase systems could potentially improve their properties. The present work looks at the subjection of Al_0.35_CoCrFeNi to AFSD and reports the resulting microstructure and mechanical behavior to better understand the strain rate sensitivity.

## 2. Materials and Methods

HEA ingots were cast from constituent elements of 99% purity using a plasma arc melting furnace to a nominal composition of Al_0.35_CoCrFeNi. The Al_0.35_CoCrFeNi alloy was selected due to its well-published baseline properties and lower hardness values for ease of deposition. Initially, individual 50 g buttons were cast and then combined and recast to form larger 100 g buttons. Each button was remelted 5 times in an ultra-high purity Argon (Ar) atmosphere to ensure elemental homogeneity of the alloying elements. The buttons were then suction cast into 12.7 mm diameter by 88.9 mm tall cylindrical rods. Energy dispersive spectroscopy (EDS) was used to assess the chemical compositions of the rods and ensured continuity between the nominal compositions and the as-cast rods as a qualitative technique previously implemented for HEAs [19]. Table 1 indicates the overall nominal compositions and the resulting EDS data. Qualitatively, the EDS results indicate that little material was lost during the process. Each rod was then annealed at 750 °C for 24 h in a steel bag and water-quenched to aid in softening the material.

**Figure 1:**
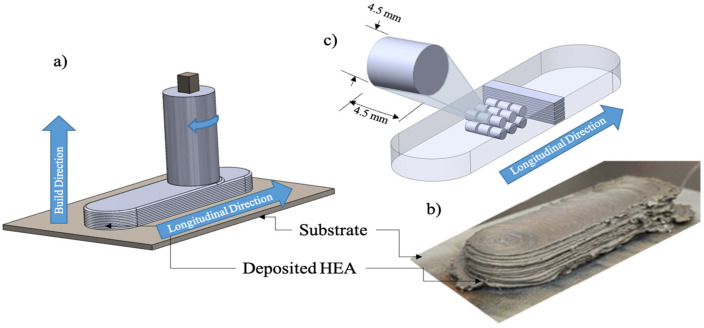
Schematic of the additive friction stir deposition process (**a**), deposited AlxCoCrFeNi high-entropy alloy (**b**), and schematic of mechanical specimen geometry and sample naming nomenclature (**c**).

The annealed Al_0.35_CoCrFeNi rods were then machined down to 7.87 × 7.87 mm rectangular feedstock pieces to be used as feedstock for the AFSD process. An aerosol graphite lubricant was applied to the rods before deposition per the manufacturer’s suggestions to prevent jamming of the feedstock in the deposition tool. A commercially available B8 AFSD machine (MELD Manufacturing, Christiansburg, VA, USA) was used to deposit the heat-treated Al_0.35_CoCrFeNi material onto a 46,100 steel substrate in a layer-by-layer approach. A 15-layer build was made with a 1 mm layer height and can be seen in Figure 1b. Figure 1a depicts the tool orientation and material orientation nomenclature used throughout this document. The build parameters used for the Al_0.35_CoCrFeNi deposition were as follows: spindle rotation speed of 500 rpm, traversing speed of 31.8 mm/min, and actuator feed rate of 27.9 mm/min. After deposition, mechanical test specimens were machined from the as-deposited material via a Mitsubishi MV1200 wire electrical discharge machine (EDM, Mitsubishi Electric Co., Ltd., Tokyo, Japan). Specimen were cut to the dimensions shown in Figure 1c for both quasi-static and intermediate-rate testing and we utilized a specimen height of 4.5 mm and a diameter of 4.5 mm.

Mechanical testing using compression was performed in the quasi-static and intermediate-strain-rate regimes. Both quasi-static and intermediate-strain-rate analyses were performed in ambient laboratory conditions using a Gleeble 3500 thermomechanical testing system (Dynamic Systems Inc., Poestenkil, NY, USA) at strain rates of 0.001 s^−1^ and 1 s^−1^ for the quasi-static and intermediate rates, respectively.

An FEI Nova NanoSEM 620 scanning electron microscope (SEM) (Thermo Fisher Scientific, Waltham, MA, USA) equipped with a Bruker (Bruker, Madison, WI, USA) energy dispersive X-ray spectroscopy (EDS) system and electron backscatter diffraction (EBSD) camera were used to collect microstructural images and chemical compositions. Multiple images across the deposited material were collected to understand the phase and grain size distributions and to explore the presence of precipitates. EDS was used to understand the chemical make-up of the phases present in both the cast heat-treated and deposited structures. Samples for metallography were prepared by mechanical grinding to a 1200 grit finish using SiC paper followed by electrolytic polishing using a solution of Struers A2 electrolyte (Struers, Copenhagen, Denmark). X-ray diffraction (XRD) analysis was used to better understand the changes in the overall phases present. XRD scans were run on a Panalytical XPert Pro X-ray diffractometer (Malvern Panalytical, Cambridge, UK) using a CoKα source at a 2-theta range of 20°–140° and a step size of 1.5°. All diffraction data were then converted to CuKα to compare them to the literature.

Microhardness was performed using a Struers Durascan Vickers hardness tester (Struers, Copenhagen, Denmark) at an indentation load of 0.5 kg. Hardness indents of the annealed structure were collected in a grid pattern of 15 × 12 indents with a spacing of 500 μm between indents. An initial hardness grid of the as-deposited Al_0.35_CoCrFeNi alloy was collected across the entirety of the specimen with a grid pattern of 46 × 31 with a 400 μm spacing.

## 3. Results and Discussion

**Figure 2:**
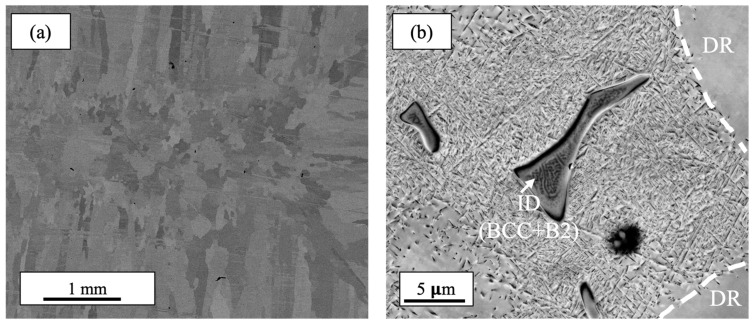
Scanning electron microscope images of the Al_0.35_CoCrFeNi HEA depicting the ID and DR regions of the microstructure (**a**) and a higher-magnification image (**b**) showing multiphase transformation products in each region.

The annealed Al_0.35_CoCrFeNi microstructure was initially studied using SEM in order to better understand the material prior to deposition. Figure 2 shows representative SEM images of the annealed alloy prior to AFSD. Macroscopically, the alloy primarily consisted of columnar grains averaging more than 1 mm in length and ~350 μm in width elongated along the solidification direction. Microstructurally, the alloy was found to consist of distinct dendritic and interdendritic regions, which transformed into a mixture of different phases either during solidification or during cooling from the annealing temperature. The dendritic region (DR) was most likely an FCC structure, whereas the interdendritic (ID) regions can be seen to display a two-phase structure of BCC and B2 formed through spinodal decomposition as the light and dark areas, respectively.

Wang et al. reported similar microstructural features for an Al_0.4_CoCrFeNi alloy. The Al_0.4_CoCrFeNi alloy displayed a mostly FCC structure with a small amount of compositional segregation [19]. Other work used in situ heating during the XRD of Al_x_CoCrFeNi (x = 0.3 and 0.5) alloys and reported a fully FCC structure for the Al_0.3_CoCrFeNi and a mixed structure for the Al_0.5_CoCrFeNi system. The Al_0.5_ system displayed a mixed structure of FCC combined with a spinodal BCC (A2) and B2 structure, leading to an FCC and ordered B2 structure [34]. TEM diffraction patterns of both the interdendritic region and the acicular precipitate revealed the same BCC (B2) structure for both. The work also found, with increasing temperatures in the Al_0.5_ system, the size of the interdendritic phases increased and BCC Al-rich precipitates developed.

**Figure 3:**
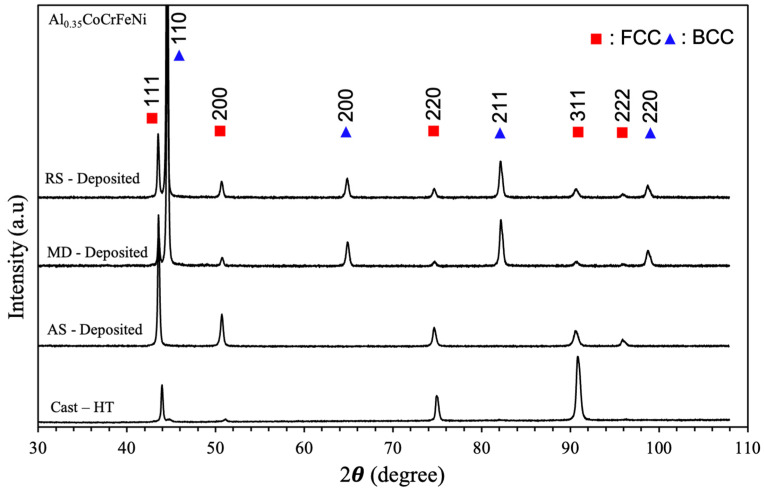
XRD patterns for the Al_0.35_CoCrFeNi high-entropy alloy for the annealed condition and then after additive friction stir deposition for the advancing side (AS), middle (MD), and retreating side (RS).

Figure 3 shows the XRD patterns for the Al_0.35_CoCrFeNi alloy. The crystal structure of the Al_x_CoCrFeNi system is widely known to be reliant on the aluminum content in the alloy. The XRD patterns shown in Figure 3 are consistent with those of systems found in the literature with an overall structure of mostly FCC with small amounts of BCC [19,20,35,36]. A small (110) reflection can be seen next to the (111) reflection. The lattice parameters were calculated to be a = 0.359 nm for the FCC peaks and a = 0.287 nm for the BCC peaks. However, the lattice parameter for BCC and that of BCC (B2) were similar and could not be discerned in the present study through XRD alone [37].

**Figure 4:**
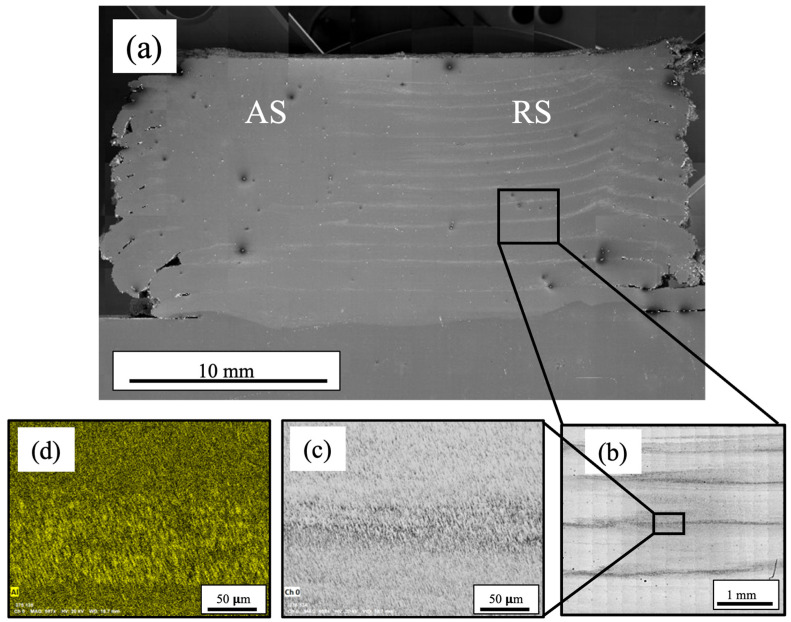
Scanning electron microscope image of additive friction stir-deposited HEA material overview (**a**), deposition layer interface (**b**,**c**), and energy dispersive spectroscopy depicting higher concentration of Al at layer interfaces (**d**).

**Figure 5:**
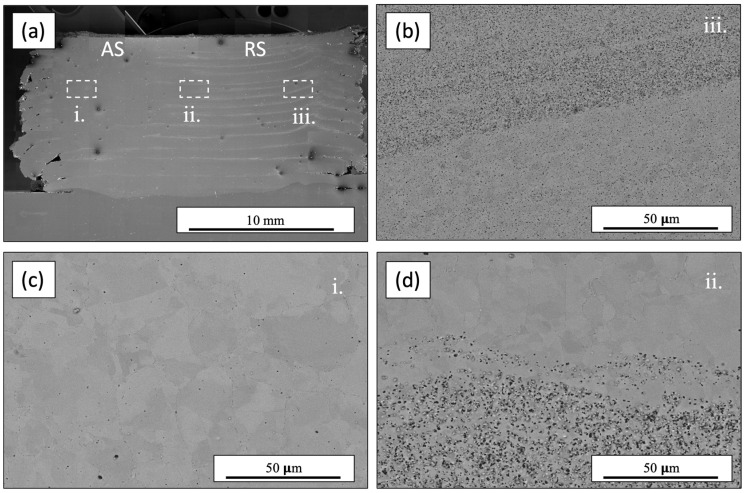
Scanning electron microscope backscatter electron images of the additive friction stir deposited overview (**a**), retreating side (**b**), advancing side (**c**), and middle (**d**).

**Figure 6:**
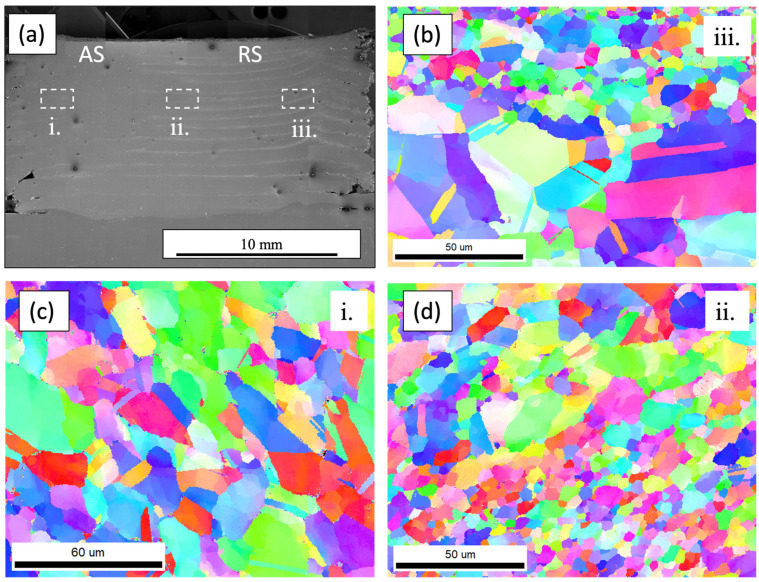
Electron backscatter diffraction inverse pole figure maps of the additive friction stir deposited overview (**a**), retreating side (**b**), advancing side (**c**), and middle (**d**). Bands of dynamically recrystallized grains can be seen in (**b**,**d**) with a significantly smaller grain size due to Zener pinning.

SEM was performed to elucidate the microstructural features present after subjecting the annealed Al_0.35_CoCrFeNi alloy to severe plasticity at elevated temperatures. Figure 4a depicts an overall cross-sectioned image machined from the deposited material with the advancing side (AS) and retreating side (RS) identified accordingly. The layer interfaces can be observed on the retreating side of the deposited material but dissipate as they approach the advancing side. Upon further investigation, the observed layer lines were strings of particles forming at the interfaces. EDS, seen in Figure 4d, revealed the particles to be Al-rich. Microstructural images captured using EBSD show a reduced grain size around the aluminum-rich particles at the layer interfaces, while the intra-layer material further from the interfaces and the Al-rich regions displayed coarser grain structures. Similar microstructures and Al-rich particle segregation was observed across two different locations along the build to confirm that features were consistent throughout. Figure 5 depicts the overall microstructure of the AFSD material along the advancing side, middle, and retreating side with a decrease in grain size observed. Figure 6 illustrates EBSD inverse pole figure (IPF) maps taken from similar locations as the BSE images in Figure 5. The EBSD data revealed a reduction in grain size proceeding from the advancing side to the retreating side. The IPF maps revealed a significant reduction in grain size along the bands of aluminum-rich particles seen on both the middle and retreating sides. The reduced grain sizes in these bands suggest Zener pinning, from the Al-rich particles, was impeding the grain growth of the dynamically recrystallized grains. Similar behavior was seen by Wang et al. in the subjugation of an Al_x_CoCrFeNi high-entropy alloy to friction stir processing. The effort utilized pockets of Al powder that were then subsequently stirred into the CoCrFeNi material. Since the friction stir process was above the melting temperature of Al, the powders first melted and then moved along the stir direction of the process and mixed with the other alloying elements. Similar to what was seen in the AFSD material, Wang et al. also observed the BCC and Al-rich phase precipitate out locally. Additionally, they reported a reduced grain size around the BCC phase present in the material system compared to the areas that were BCC and Al-deficient [38]. Reduced grain sizes due to Zener pinning have also been reported in NiCoCr medium-entropy alloys [39]. Jodi et al. reported that a distribution of finer Cr_2_N precipitates contributed more to the reduction in grain size from Zener pinning pressure than the coarser lower fraction of Cr_2_N [40]. Similarly, Agrawal et al. observed a significant reduction in the grain size of an Fe_40_Mn_20_Co_20_Cr_15_Si_5_ (at%) HEA subjected to AFSD due to discontinuous dynamic recrystallization. Agrawal et al. also observed an increase in the grain size from alterations in the processing parameter increasing the heat generated and subsequently affecting the mechanical properties [31].

**Figure 7:**
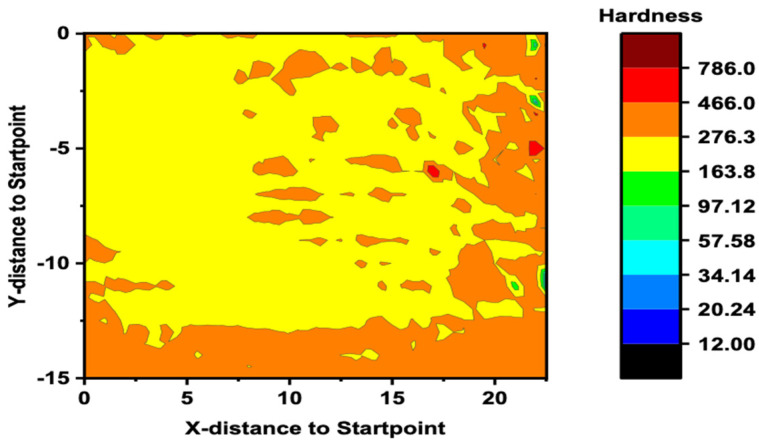
Microhardness map of the Al_0.35_CoCrFeNi alloy system deposited via additive friction stir deposition. Overall map with ~500 μm spacing between indents.

To further understand these regions, microhardness tests were performed on the entire cross section of the deposit. The microhardness grid revealed a similarly layered structure to that of the SEM backscatter image with a significantly increased hardness at the locations with high concentrations of Al-rich particles, as can be observed in Figure 7. The increased hardness was likely from a mix of decreased grain size, due to Zener pinning, as well as the presence of a BCC phase due to the higher concentrations of Al. Significant work has been performed in the literature displaying the effects of Al content on the breakdown of FCC and BCC phases in AlCoCrFeNi composition. Kao et al. reported the increase in hardness and the shift from a predominantly FCC structure to a split FCC/BCC and then to a predominantly BCC structure with increasing Al content [20]. Similarly, Joseph et al. studied the increase in Al content in AlCoCrFeNi alloys through the use of nanoindentation and observed an increase in nano-hardness in the BCC structure over that in the FCC structure [41]. The individual layers deposited can be observed for both the middle and RS locations with significant increases in hardness leading to the Al-rich locations and then decreasing again when moving away. The average annealed hardness was found to be ~175 HV as a comparison.

X-ray diffraction revealed a significant shift in the phase content compared to that in the original feedstock material. Figure 3 illustrates the XRD patterns gathered of the feedstock as well as the AS and RS locations on the deposited material. The advancing side of the deposit contained a fully FCC structure, whereas the RS location exhibited a dual phase FCC/BCC structure due to the higher Al content present at the layer interfaces. The (110) reflection of the BCC structure, which was previously observed in the initial feedstock material, significantly increased in the RS location of the deposit. Reflection peaks for (200), (211), and (220) in the BCC structure can also be seen for the RS location of the deposit. The changes in phase content between the initial cast material and the AS and RS of the deposited material were most likely caused by a combination of a dispersion of aluminum in the deposited material and a change in cooling rates between the two processes. Chen et al. reported that varying cooling rates in CrFeCoNiAl_0.6_ can produce ultra-fine FCC and BCC phases. The work also demonstrated the ability to adjust the FCC and BCC content to tailor mechanical strength based on the cooling rate [42]. The work by Chen et al. suggests further tailoring of the microstructural and mechanical properties of the deposited material could be achieved.

**Figure 8:**
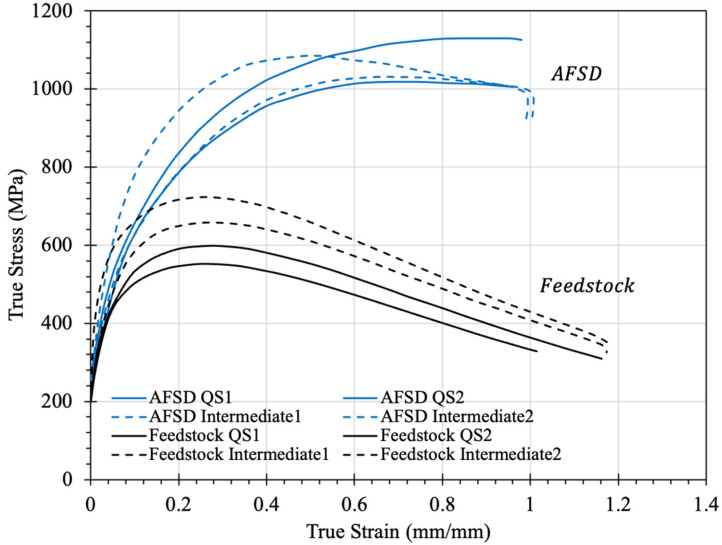
Stress–strain response of annealed and additive friction stir-deposited Al_0.35_CoCrFeNi evaluated using compression at 0.001 s^−1^ (QS) and 1 s^−1^ (intermediate) strain rates.

Compression was run on both the feedstock and AFSD material to better understand the change in mechanical performance from the annealed state to that of the severely plasticly deformed material. Compression was performed at quasi-static and intermediate strain rates of 0.001 s^−1^ and 1 s^−1^, respectively. Figure 8 depicts the overall stress–strain behavior of the two different material states. An obvious increase in the strength of the AFSD material can be seen with a change in the maximum stress of 576 MPa and 1074 Mpa for the feedstock and AFSD materials, respectively, at the 0.001 s^−1^ strain rate. A similar increase in maximum stress for the 1 s^−1^ strain rate can be seen. The increase in overall strength can be attributed to both the Hall–Petch effect, from a decrease in the grain size, and a split FCC + BCC structure compared to the mostly FCC structure observed in the feedstock material.

The feedstock material also exhibited a strain rate sensitivity when increasing from the 0.001 s^−1^ strain rate to that of the 1 s^−1^. Positive strain rate sensitivity for peak stress and yield stress could be due to the higher rate of increasing the barriers of dislocation motion for mechanically and thermally activated deformations. Another cause could be the higher strain rate decelerating the dislocation annihilation [43]. An increase in maximum stress from an average of 575 MPa to 690 MPa was observed for the 0.001 s^−1^ and 1 s^−1^ rates, respectively. However, the AFSD material did not exhibit the same trend. A larger scatter can be observed in the AFSD material’s stress–strain behavior with maximum stresses overlapping for the 0.001 s^−1^ and 1 s^−1^ rates and averages of 1074 MPa and 1057 MPa, respectively. The cause of the apparent strain rate insensitivity could be due to the placement of samples taken in the AFSD deposit. Material from both the AS and RS portions of the deposit were used in the mechanical testing. However, Figure 7 shows an obvious discrepancy in the overall hardness of the deposit when proceeding from the retreating side to the advancing side. The change in local mechanical properties could be contributing to the variation in the overall response.

**Figure 9:**
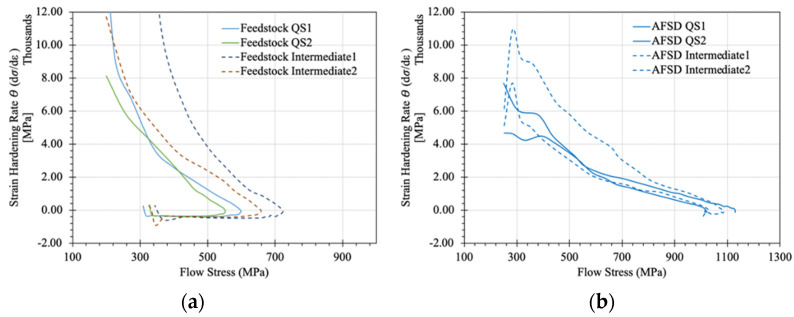
Kocks–Mecking plot showing variation in dσ/dε as a function of flow stress for the (**a**) annealed HEA material and (**b**) additive friction stir-deposited (AFSD) HEA material evaluated using compression at 0.001 s^−1^ (QS) and 1 s^−1^ (intermediate) strain rates.

The overall strain hardening behavior of the feedstock and AFSD material was also studied. Figure 9 illustrates Kocks–Mecking plots showing the variation in dσ/dε as a function of flow stress for the two material systems. An obvious change in the overall material behavior can be observed, with the feedstock material having a significant amount of softening present in the material behavior when compared to that of the AFSD system. At the initial onset of the AFSD material tested at 0.001 s^−1^, the strain hardening rate, θ, decreased linearly, which is characteristic of stage-III hardening. The inflection point that the linear region ended at is also representative of the critical stress in the material system. After the inflection point, the work hardening rate decreased significantly due to dynamic recrystallization and the onset of stage-IV hardening [44]. In contrast, the strain hardening behavior of the feedstock material neither showed a linear region nor an inflection point indicative of the dynamic recrystallization (DRX) seen in the AFSD material. The system did, however, exhibit a pronounced peak stress with an obvious softening behavior indicative of resistance to DRX. Sluggish diffusion behavior is reported as the culprit for AlCoCrFeNi alloy systems’ resistance to recrystallization [45,46,47].

## 4. Conclusions

This investigation is the first work to look at a solid-state additive-manufactured HEA microstructures and mechanical behaviors, specifically the Al_0.35_CoCrFeNi HEA processed by AFSD. Overall, the work found that processing HEA materials through AFSD, could prove to be an effective manufacturing methodology when increasing strength from further alloying is unavailable. The effort found the following specific conclusions:

- The annealed Al_0.35_CoCrFeNi system exhibited a mostly FCC structure with small pockets of BCC phases.
- Deposition of the Al_0.35_CoCrFeNi via AFSD revealed a variation in the phase content in the retreating side versus the advancing side due to a gradient in the temperatures present during the process.
- Electron backscatter diffraction revealed an increase in Al-rich particles along the layer interfaces of the retreating side that potentially impeded grain growth through Zener pinning and increased the overall BCC phase structure present along the retreating side.
- The overall strength of the deposited material was approximately doubled compared to that of the cast material system with increases in strength at both quasi-static and intermediate strain rates due to the Hall–Petch effect and a split FCC + BCC phase structure.
- A positive strain-rate sensitivity in the AFSD material was observed, with the intermediate-rate material exhibiting a yield strength approximately twice that of the quasi-static tested material.
- Further mechanical performance could be gained from the implementation of a post-deposition heat treatment to dissolve the Al-rich particles and further increase the BCC structure.

## Figures and Tables

**Table 1:** Chemical composition of Al_0.35_CoCrFeNi high-entropy alloy system.

	Elements	Al	Co	Cr	Fe	Ni
Nominal	at.%	8	23	23	23	23
Measured	at.%	8.05 ± 0.18	23.72 ± 0.7	22.72 ± 0.58	23.23 ± 0.63	22.28 ± 0.63

## Data Availability

The data presented in this study are available on request from the corresponding author.

## References

[1] Yeh J.-W., Lin S.-J. (2018). Breakthrough Applications of High-Entropy Materials. J. Mater. Res..

[2] Tsao T.-K., Yeh A.-C., Kuo C.-M., Kakehi K., Murakami H., Yeh J.-W., Jian S.-R. (2017). The High Temperature Tensile and Creep Behaviors of High Entropy Superalloy. Sci. Rep..

[3] Padture N.P., Gell M., Jordan E.H. (2002). Thermal Barrier Coatings for Gas-Turbine Engine Applications. Science.

[4] Yan X., Zhang Y. (2020). Functional Properties and Promising Applications of High Entropy Alloys. Scr. Mater..

[5] Praveen S., Kim H.S. (2018). High-Entropy Alloys: Potential Candidates for High-Temperature Applications—An Overview. Adv. Eng. Mater..

[6] Jia Z., Nomoto K., Wang Q., Kong C., Sun L., Zhang L., Liang S., Lu J., Kruzic J.J. (2021). A Self-Supported High-Entropy Metallic Glass with a Nanosponge Architecture for Efficient Hydrogen Evolution under Alkaline and Acidic Conditions. Adv. Funct. Mater..

[7] Zhang Y., Zuo T.T., Tang Z., Gao M.C., Dahmen K.A., Liaw P.K., Lu Z.P. (2014). Microstructures and Properties of High-Entropy Alloys. Prog. Mater. Sci..

[8] Miracle D.B., Senkov O.N. (2017). A Critical Review of High Entropy Alloys and Related Concepts. Acta Mater..

[9] Zhang Y., Peng W. (2012). jie Microstructural Control and Properties Optimization of High-Entrop Alloys. Procedia Eng..

[10] Ye Y.F., Wang Q., Lu J., Liu C.T., Yang Y. (2016). High-Entropy Alloy: Challenges and Prospects. Mater. Today.

[11] Zou Y., Ma H., Spolenak R. (2015). Ultrastrong Ductile and Stable High-Entropy Alloys at Small Scales. Nat. Commun..

[12] Yeh J.-W., Chen S.-K., Lin S.-J., Gan J.-Y., Chin T.-S., Shun T.-T., Tsau C.-H., Chang S.-Y. (2004). Nanostructured High-Entropy Alloys with Multiple Principal Elements: Novel Alloy Design Concepts and Outcomes. Adv. Eng. Mater..

[13] Li Z., Pradeep K.G., Deng Y., Raabe D., Tasan C.C. (2016). Metastable High-Entropy Dual-Phase Alloys Overcome the Strength–Ductility Trade-Off. Nature.

[14] Diao H.Y., Feng R., Dahmen K.A., Liaw P.K. (2017). Fundamental Deformation Behavior in High-Entropy Alloys: An Overview. Curr. Opin. Solid State Mater. Sci..

[15] Joseph J., Stanford N., Hodgson P., Fabijanic D.M. (2017). Tension/Compression Asymmetry in Additive Manufactured Face Centered Cubic High Entropy Alloy. Scr. Mater..

[16] Zhou Y.J., Zhang Y., Kim T.N., Chen G.L. (2008). Microstructure Characterizations and Strengthening Mechanism of Multi-Principal Component AlCoCrFeNiTi0.5 Solid Solution Alloy with Excellent Mechanical Properties. Mater. Lett..

[17] Raabe D., Tasan C.C., Springer H., Bausch M. (2015). From High-Entropy Alloys to High-Entropy Steels. Steel Res. Int..

[18] Li Z., Raabe D. (2018). Influence of Compositional Inhomogeneity on Mechanical Behavior of an Interstitial Dual-Phase High-Entropy Alloy. Mater. Chem. Phys..

[19] Wang W.-R., Wang W.-L., Wang S.-C., Tsai Y.-C., Lai C.-H., Yeh J.-W. (2012). Effects of Al Addition on the Microstructure and Mechanical Property of AlxCoCrFeNi High-Entropy Alloys. Intermetallics.

[20] Kao Y.-F., Chen T.-J., Chen S.-K., Yeh J.-W. (2009). Microstructure and Mechanical Property of As-Cast, -Homogenized, and -Deformed AlxCoCrFeNi (0 ≤ x ≤ 2) High-Entropy Alloys. J. Alloys Compd..

[21] Shiratori H., Fujieda T., Yamanaka K., Koizumi Y., Kuwabara K., Kato T., Chiba A. (2016). Relationship between the Microstructure and Mechanical Properties of an Equiatomic AlCoCrFeNi High-Entropy Alloy Fabricated by Selective Electron Beam Melting. Mater. Sci. Eng. A.

[22] Niu P.D., Li R.D., Yuan T.C., Zhu S.Y., Chen C., Wang M.B., Huang L. (2019). Microstructures and Properties of an Equimolar AlCoCrFeNi High Entropy Alloy Printed by Selective Laser Melting. Intermetallics.

[23] Brif Y., Thomas M., Todd I. (2015). The Use of High-Entropy Alloys in Additive Manufacturing. Scr. Mater..

[24] Chen S., Tong Y., Liaw P. (2018). Additive Manufacturing of High-Entropy Alloys: A Review. Entropy.

[25] Li C., Ferry M., Kruzic J.J., Li X. (2022). Review: Multi-Principal Element Alloys by Additive Manufacturing. J. Mater. Sci..

[26] Rivera O.G., Allison P.G., Jordon J.B., Rodriguez O.L., Brewer L.N., McClelland Z., Whittington W.R., Francis D., Su J., Martens R.L. (2017). Microstructures and Mechanical Behavior of Inconel 625 Fabricated by Solid-State Additive Manufacturing. Mater. Sci. Eng. A.

[27] Su J.-Q., Nelson T.W., McNelley T.R., Mishra R.S. (2011). Development of Nanocrystalline Structure in Cu during Friction Stir Processing (FSP). Mater. Sci. Eng. A.

[28] Avery D.Z., Phillips B.J., Mason C.J.T., Palermo M., Williams M.B., Cleek C., Rodriguez O.L., Allison P.G., Jordon J.B. (2020). Influence of Grain Refinement and Microstructure on Fatigue Behavior for Solid-State Additively Manufactured Al-Zn-Mg-Cu Alloy. Met. Mater. Trans. A.

[29] Phillips B.J., Avery D.Z., Liu T., Rodriguez O.L., Mason C.J.T., Jordon J.B., Brewer L.N., Allison P.G. (2019). Microstructure-Deformation Relationship of Additive Friction Stir-Deposition Al–Mg–Si. Materialia.

[30] Yu H.Z. (2022). Additive Friction Stir Deposition.

[31] Agrawal P., Haridas R.S., Agrawal P., Mishra R.S. (2022). Deformation Based Additive Manufacturing of a Metastable High Entropy Alloy via Additive Friction Stir Deposition. Addit. Manuf..

[32] Mason C.J.T., Rodriguez R.I., Avery D.Z., Phillips B.J., Bernarding B.P., Williams M.B., Cobbs S.D., Jordon J.B., Allison P.G. (2021). Process-Structure-Property Relations for as-Deposited Solid-State Additively Manufactured High-Strength Aluminum Alloy. Addit. Manuf..

[33] Rutherford B.A., Avery D.Z., Phillips B.J., Rao H.M., Doherty K.J., Allison P.G., Brewer L.N., Jordon J.B. (2020). Effect of Thermomechanical Processing on Fatigue Behavior in Solid-State Additive Manufacturing of Al-Mg-Si Alloy. Metals.

[34] Wang W.-R., Wang W.-L., Yeh J.-W. (2014). Phases, Microstructure and Mechanical Properties of AlxCoCrFeNi High-Entropy Alloys at Elevated Temperatures. J. Alloys Compd..

[35] Li C., Li J.C., Zhao M., Jiang Q. (2010). Effect of Aluminum Contents on Microstructure and Properties of AlxCoCrFeNi Alloys. J. Alloys Compd..

[36] Yang T., Xia S., Liu S., Wang C., Liu S., Zhang Y., Xue J., Yan S., Wang Y. (2015). Effects of AL Addition on Microstructure and Mechanical Properties of Al CoCrFeNi High-Entropy Alloy. Mater. Sci. Eng. A.

[37] Tang Z., Senkov O.N., Parish C.M., Zhang C., Zhang F., Santodonato L.J., Wang G., Zhao G., Yang F., Liaw P.K. (2015). Tensile Ductility of an AlCoCrFeNi Multi-Phase High-Entropy Alloy through Hot Isostatic Pressing (HIP) and Homogenization. Mater. Sci. Eng. A.

[38] Wang T., Shukla S., Komarasamy M., Liu K., Mishra R.S. (2019). Towards Heterogeneous AlxCoCrFeNi High Entropy Alloy via Friction Stir Processing. Mater. Lett..

[39] Hu G.W., Zeng L.C., Du H., Wang Q., Fan Z.T., Liu X.W. (2021). Combined Effects of Solute Drag and Zener Pinning on Grain Growth of a NiCoCr Medium-Entropy Alloy. Intermetallics.

[40] Jodi D.E., Park J., Park N. (2019). Precipitate Behavior in Nitrogen-Containing CoCrNi Medium-Entropy Alloys. Mater. Charact..

[41] Joseph J., Stanford N., Hodgson P., Fabijanic D.M. (2017). Understanding the Mechanical Behaviour and the Large Strength/Ductility Differences between FCC and BCC AlxCoCrFeNi High Entropy Alloys. J. Alloys Compd..

[42] Chen C., Fan Y., Wang W., Zhang H., Hou J., Wei R., Zhang T., Wang T., Li M., Guan S. (2022). Synthesis of Ultrafine Dual-Phase Structure in CrFeCoNiAl0.6 High Entropy Alloy via Solid-State Phase Transformation during Sub-Rapid Solidification. J. Mater. Sci. Technol..

[43] Wang W., Ma Y., Yang M., Jiang P., Yuan F., Wu X. (2017). Strain Rate Effect on Tensile Behavior for a High Specific Strength Steel: From Quasi-Static to Intermediate Strain Rates. Metals.

[44] Annasamy M., Haghdadi N., Taylor A., Hodgson P., Fabijanic D. (2019). Dynamic Recrystallization Behaviour of AlxCoCrFeNi High Entropy Alloys during High-Temperature Plane Strain Compression. Mater. Sci. Eng. A.

[45] Tsai C.-W., Chen Y.-L., Tsai M.-H., Yeh J.-W., Shun T.-T., Chen S.-K. (2009). Deformation and Annealing Behaviors of High-Entropy Alloy Al0.5CoCrCuFeNi. J. Alloys Compd..

[46] Ng C., Guo S., Luan J., Shi S., Liu C.T. (2012). Entropy-Driven Phase Stability and Slow Diffusion Kinetics in an Al0.5CoCrCuFeNi High Entropy Alloy. Intermetallics.

[47] Wu Z., Bei H., Otto F., Pharr G.M., George E.P. (2014). Recovery, Recrystallization, Grain Growth and Phase Stability of a Family of FCC-Structured Multi-Component Equiatomic Solid Solution Alloys. Intermetallics.

